# A prospective study of surgery and adjuvant chemotherapy for primary gastric lymphoma stage II.

**DOI:** 10.1038/bjc.1997.582

**Published:** 1997

**Authors:** T. Takenaka, K. Maruyama, T. Kinoshita, M. Sasako, T. Sano, H. Katai, Y. Matsuno

**Affiliations:** Department of Medical Oncology, National Cancer Center Hospital, Tokyo, Japan.

## Abstract

**Images:**


British Journal of Cancer (1997) 76(11), 1484–1488
© 1997 Cancer Research Campaign

### A prospective study of surgery and adjuvant chemotherapy for primary gastric lymphoma stage II

**T Takenaka[1], K Maruyama[2], T Kinoshita[3], M Sasako[2], T Sano[2], H Katai[2] and Y Matsuno[4]**

Departments of [1]Medical Oncology and [2]Surgical Oncology, National Cancer Center Hospital, Tokyo, Japan; [3]Department of Surgical Oncology, National Cancer Center Hospital East, Chiba, Japan; [4]Pathology Division, National Cancer Center Research Institute, Tokyo, Japan

**Summary** The standard management of primary gastric lymphoma (PGL) (stage II) has not been established despite the use of various treatment modalities. The present prospective trial of combined surgery and chemotherapy for the treatment of PGL (stage II) included 25 consecutive patients treated between July 1978 and December 1993. Twenty-one patients were treated with total gastrectomy and four with partial gastrectomy; this was followed by post-operative chemotherapy with m-VEPA (vincristine, cyclophosphamide, prednisolone and doxorubicin), followed by consolidation chemotherapy with VEMP (vindesine, cyclophosphamide, methotrexate and prednisolone) or VQEP (vindesine, carbazilquinone, cyclophosphamide and prednisolone). Twenty-one of the 25 patients who completed post-operative chemotherapy were free of relapse 26–203 (median 94) months after the gastrectomy. Of the four patients who did not complete the projected chemotherapy, two relapsed and died of lymphoma. Another patient with recurrent lymphoma died in an accident, and the fourth patient was in remission at 54 months after surgery. The post-operative overall and disease-free survival rates at 10 years for the 25 evaluable patients were 81.6% and 92.0% respectively. Major surgical complications and treatment-related death after chemotherapy were not observed. PGL (stage II) appears to be curable when treated with gastrectomy and adjuvant chemotherapy.

**Keywords:** primary gastric lymphoma; adjuvant chemotherapy

The stomach is the most common site of extranodal lymphomas, accounting for about 24% of cases (Freeman et al, 1972). Although localized (stage I and II) primary gastric lymphoma (PGL) has been treated by various modalities, including surgery (Shiu et al, 1982), chemotherapy (Maor et al, 1984: Salles et al, 1991), surgery plus radiotherapy (Shiu et al, 1982; Gospodarowicz et al, 1983; Taal et al, 1993), surgery plus chemotherapy (Paulson et al, 1983; Sheridan et al, 1985; Shepherd et al, 1988; Bellesi et al, 1989; Pasini et al, 1994) and radiotherapy plus chemotherapy (Burgers et al, 1988; Dragosics et al, 1985, Maor et al, 1990; Tondini et al, 1993), the best management for this disease is still unclear.

During the last two decades, many combination chemotherapy regimens including doxorubicin (McKelvey et al, 1976; Rodriguez et al, 1977; Skarin et al, 1977) were developed for patients with aggressive non-Hodgkin's lymphoma (NHL), and the treatment of advanced-stage NHL patients has been one of the major successes of cancer therapy.

A combination chemotherapy known as VEM(N)P [vincristine, cyclophosphamide (Endoxan), 6-mercaptopurine or procarbazine, and prednisolone (Sakai, 1976)] was regarded in Japan around 1975 as a standard regimen for patients with advanced malignant lymphoma. At that time, we treated two patients with PGL (stage II) with a 3-year cyclic VEMP therapy after surgical resection; they survived without relapse for over 9 years (Takenaka et al, 1981).

The Japanese Lymphoma Study Group began a prospective study of advanced NHL patients treated with VEPA chemotherapy (vincrinstine, cyclophosphamide, prednisolone and doxorubicin) (Lymphoma Study Group, 1979; Shimoyama et al, 1988) in 1978. In that trial, consolidation chemotherapy using various regimens, including VEMP (vindesine, cyclophosphamide, methotrexate and prednisolone) and VQEP (vindesine, carbazilquinone, cyclophosphamide and prednisolone), was planned to continue monthly for at least 2 years. In our pilot study of six advanced and/or non-resectable PGL patients treated with a VEPA-like regimen, a complete response was observed in 50% and partial response in 17% of the patients (Takenaka et al, 1982).

To avoid the problems associated with the interpretation of the wide variation in results obtained using different treatment approaches in retrospective studies, we began a prospective trial of combined surgery and chemotherapy for the treatment of PGL (stage II) patients in 1978. The results are presented here.

### PATIENTS AND METHODS

### Patients

Sixty-seven patients were diagnosed as having PGL [defined according to the modified criteria of Dawson (1961)] and 61 of these patients (91%) underwent gastrectomy at the NCC Hospital between July 1978 and December 1993. Twenty-nine patients were diagnosed as having stage II disease according to the Ann Arbor classification (Carbone et al, 1971). Of these patients, 25 agreed to enter into the trial.

*Received 29 October 1996*
*Revised 13 May 1997*
*Accepted 21 May 1997*

*Correspondence to:* T Takenaka, Department of Medical Oncology, National Cancer Center Hospital, 1-1 Tsukiji 5-chome, Chuo-ku, Tokyo 104, Japan

**Table 1** Chemotherapy regimens administered to 25 patients with primary gastric lymphoma (stage II) (1978–93)

| Regimen | Dose | Route | Days given |
|---|---|---|---|
| m-VEPA (every 28 days) | | | |
| Vincristine | 1 mg $m^{-2}$ | i.v. | 1, 8 |
| Endoxan (cyclophosphamide) | 350 mg $m^{-2}$ | i.v. | 1, 8 |
| Prednisolone | 30 mg $m^{-2}$ | p.o. | 1–3, 8–10 |
| Doxorubicin | 30 mg $m^{-2}$ | i.v. | 1 |
| VEMP (every 28 days) | | | |
| Vindesine | 2 mg $m^{-2}$ | i.v. | 1, 8 |
| Endoxan (cyclophosphamide) | 350 mg $m^{-2}$ | i.v. | 1, 8 |
| Methotrexate | 30 mg $m^{-2}$ | i.v. | 1, 8 |
| Prednisolone | 30 mg $m^{-2}$ | p.o | 1–3, 8–10 |
| VQEP (every 28 days) | | | |
| Vindesine | 2 mg $m^{-2}$ | i.v. | 1, 8 |
| Carbazilquinone | 2 mg $m^{-2}$ | i.v. | 1, 8 |
| Endoxan (cyclophosphamide) | 350 mg $m^{-2}$ | i.v. | 1, 8 |
| Prednisolone | 30 mg $m^{-2}$ | p.o. | 1–3, 8–10 |

**Figure 1** Overall survival curve (**A**) and disease-free survival curve (**B**) in 25 primary gastric lymphoma (stage II) patients treated with gastrectomy followed by chemotherapy. Tick marks indicate the date on which the patient was last examined (alive)

### Histopathology

All biopsy and surgical specimens, originally diagnosed according to the Working Formulation (WF) of non-Hodgkin's lymphoma (The Non-Hodgkin's Lymphoma Pathologic Classification Project, 1982).

In addition, surgical specimens were re-evaluated if histological features indicating an origin from mucosa-associated lymphoid tissue (MALT) (Isaacson et al, 1983) were present and classified into the following three groups: MALT lymphoma with or without areas of large-cell cytology (low-grade MALT); diffuse large-cell lymphoma with areas showing MALT features (high-grade MALT); and diffuse large-cell lymphoma without MALT features (non-MALT).

### Staging

The clinicopathological stages of the 25 patients were diagnosed and determined from the physical, radiological, endoscopical, surgical and histopathological findings, including routine haematological and chemical examinations, bone marrow aspiration and renal function test. The examinations used for the staging varied at times. Chest radiography and gastrointestinal contrast radiography were performed in all 25 patients; $^{67}$Ga scintigraphy of the total body was performed in 23 patients and ultrasonography of the abdomen in 24 patients. Only seven patients in this series were examined by computerized tomography (CT). Consequently, patients were staged according to the original Ann Arbor classification in the present study.

### Chemotherapy

Chemotherapy according to the modified-VEPA (m-VEPA) regimen was given as post-operative chemotherapy for about 1 year to all 25 of these stage II PGL patients with no evidence of macroscopic residual disease (Table 1).

Additional chemotherapy ('consolidation' chemotherapy), according to the VEMP regimen or the VQEP regimen was given mainly to patients with no evidence of active disease after about 1 year of post-operative chemotherapy (Table 1).

### Statistics

Survival was calculated from the date of surgery to the last follow-up or to the date of death. The last follow-up date was August 1996. Survival curves were plotted using the Kaplan–Meier method (Kaplan and Meier, 1958).

### RESULTS

### Surgery and clinicopathological findings

Radical tumour resection with curative intent was performed in 61 consecutive patients with PGL. Of the 29 patients diagnosed as having stage II disease, three patients elected to be treated by second-generation chemotherapy as post-operative chemotherapy and another patient refused adjuvant treatment.

The remaining 25 PGL (stage II) patients were enrolled in the study (Table 2). Fourteen patients were women and 11 were men. Their ages at presentation ranged from 36 to 82 (median 56) years. In 4 of the 25 patients, partial gastrectomy was performed; total gastrectomy was performed in the other 21.

The size of the primary tumour varied from 3.4 to 20.0 (median 10.5) cm in the maximum diameter. Twenty patients were classified as having diffuse large cell lymphoma, three as mixed small and large cell, one as diffuse small cleaved cell and one as follicular predominantly large cell lymphoma. All patients were classified as having intermediate-grade lymphoma (WF-system).

Three of the 22 re-evaluated patients were classified as having low-grade MALT lymphoma and 13 patients as having high-grade MALT lymphoma. The remaining six patients were classified as having non-MALT lymphoma.

The depth of tumour infiltration into the gastric wall was the submucosa in seven patients, muscularis propria in five, subserosa in five and serosa without the involvement of adjacent organs in eight.

© Cancer Research Campaign 1997

**Table 2** Patients with stage II primary gastric lymphoma

| Patient number | Sex/ Age | Working formulation | MALT grade | Gastrectomy | Size (cm) | Tumour depth | Node invasion | Resection margin | Post-operative chemotherapy (courses) | Additional chemotherapy (courses) | Relapse (months) | Survival (months) | Status |
|---|---|---|---|---|---|---|---|---|---|---|---|---|---|
| 1 | M/41 | DL | High | Total | 14.0 | S | 8/33 | + (aw) | 12 | 8 | – | 203 | A |
| 2 | F/36 | DL | NE | Total | 7.0 | SS | 1/55 | – | 11 | 10 | – | 170 | A |
| 3 | M/73 | DL | NE | Total | 14.0 | S | 6/57 | – | 12 | 9 | – | 167 | A |
| 4 | M/41 | DL | NE | Partial | 4.8 | P M | 4/68 | – | 14 | 6 | – | 127 | A |
| 5 | M/72 | DL | High | Total | 12.5 | S M | 19/38 | – | 3 | – | 6 | 8 | D |
| 6 | F/44 | DL | None | Total | 8.0 | P M | 1/30 | – | 13 | 11 | – | 120 | A |
| 7 | F/55 | DL | None | Partial | 9.0 | S | 1/29 | – | 15 | 10 | – | 121 | A |
| 8 | M/51 | DL | High | Total | 14.0 | P M | 3/32 | – | 12 | 12 | – | 113 | A |
| 9 | F/51 | DL | None | Total | 14.0 | P M | 3/36 | – | 12 | 12 | – | 109 | A |
| 10 | M/44 | DL | High | Total | 13.0 | S S | 1/34 | – | 12 | 11 | – | 102 | A |
| 11 | F/65 | DL | High | Total | 15.0 | S M | 1/55 | – | 14 | 8 | – | 100 | A |
| 12 | F/76 | DL | None | Partial | 6.0 | S | 3/16 | – | 13 | 9 | – | 29 | D* |
| 13 | M/59 | DL | High | Total | 13.5 | S | 10/104 | – | 12 | 14 | – | 94 | A |
| 14 | F/64 | DSC | High | Total | 3.4 | S M | 7/89 | – | 4 | – | 15 | 35 | D** |
| 15 | F/58 | DL | None | Total | 10.5 | S S | 4/62 | – | 4 | – | – | 57 | D* |
| 16 | F/47 | DL | High | Total | 6.0 | S M | 13/93 | – | 12 | 7 | – | 75 | A |
| 17 | M/53 | DM | High | Total | 20.0 | S | 13/78 | – | 12 | 10 | – | 79 | A |
| 18 | F/82 | DL | Low | Total | 10.5 | P M | 4/81 | – | 10 | 10 | – | 70 | A |
| 19 | M/49 | FL | None | Total | 6.0 | S M | 2/44 | – | 3 | – | – | 54 | A |
| 20 | F/73 | DM | Low | Total | 10.5 | S | 5/123 | – | 11 | – | – | 55 | A |
| 21 | M/59 | DM | High | Total | 11.0 | S S | 1/91 | – | 12 | – | – | 48 | A |
| 22 | F/65 | DL | Low | Total | 15.0 | S | 3/64 | – | 10 | 6 | – | 34 | A |
| 23 | M/56 | DL | High | Total | 13.0 | S M | 2/33 | + (ow) | 11 | – | – | 47 | A |
| 24 | F/57 | DL | High | Partial | 8.0 | S M | 1/85 | – | 12 | – | – | 36 | A |
| 25 | F/54 | DL | High | Total | 6.6 | S S | 5/39 | – | 10 | – | – | 26 | A |

DL, diffuse large cell; DSC, diffuse small cleaved cell; DM, diffuse mixed small and large cell; NE, not evaluated; FL, follicular predominantly large cell; SM, submucosa; PM, muscularis propria, SS, subserosa; S, serosa; A, alive; D, died of malignant lymphoma (ML); D, died of other than ML, D* died of ML + other.

The number of lymph nodes involved ranged from 1 to 19 (median 3). In two patients, there was microscopic involvement of the resection margin with lymphoma cells.

### Chemotherapy and prognosis

Twenty-four patients were treated with m-VEPA and one patient was treated with the same regimen excluding doxorubicin because of her advanced age (82 years).

Twenty-one of the 25 patients completed the post-operative chemotherapy (m-VEPA), which ranged from 10 to 15 courses (median 12 courses). Three out of five patients who received more than 12 courses of m-VEPA were administered reduced doses of doxorubicin, which ranged from 90% to 80% per course, according to the judgement of the attending physicians. The remaining two patients were treated with reduced doses (90% and 85%) of vincristine and doxorubicin per course because of their potential for adverse effects. Consequently, one to three courses of m-VEPA were supplementally administered to these patients. Of these 21 patients, 16 received consolidation chemotherapy ranging from 6 to 14 (median 10) courses; the other five patients refused consolidation chemotherapy. One patient was treated with 14 courses of consolidation chemotherapy by the judgement of his doctor.

Four of the 25 patients could not complete even the projected post-operative chemotherapy. There was one death from tumour recurrence 8 months after surgery. In this patient, chemotherapy was terminated after only three courses of m-VEPA because of severe liver dysfunction induced by reactivated *Schistosomiasis japonica*. In the other three patients, treatment was limited to three or four courses of m-VEPA at the patients' request. One patient with tumour relapse died in an accident and another patient died of pulmonary disease without any evidence of lymphoma. The remaining patient has continued in complete remission 54 months after surgery.

The median overall survival rate has not yet been reached between 26 and 203 months after gastrectomy. The overall survival rate for all 25 patients was 81.6% at 10 years, including all causes of death (Figure 1A). Of interest is the finding that the disease-free survival period showed a long plateau (excluding the two patients with early treatment failure or accidental death). The disease-free survival rate was 92.0% at 10 years (Figure 1B). Of the 21 patients who received more than ten courses of post-operative chemotherapy, none developed recurrence.

### Toxicity and complications

No major surgical complications, such as operative mortality, bleeding and dumping syndrome disturbing daily lives or treatment-related death after chemotherapy, were observed in this series. However, loss of weight (about 10%) occurred in almost all of the patients during chemotherapy.

Chemotherapy was complicated in two patients by moderate peripheral neuropathy; their vincristine doses were reduced. Three patients developed a reactivation of chronic hepatitis B or C virus because of chemotherapy; their treatment was stopped for several weeks. Two documented infections were observed in two patients: one patient developed mycoplasma pneumonia and the other enterococcal septicaemia. Although leucocytopenia ($< 2.0 \times 10^3\ \mu l^{-1}$) was observed during the post-operative or

© Cancer Research Campaign 1997

consolidation chemotherapy in 8 of the 25 patients, no thrombocytopenia ($< 10 \times 10^4\ \mu l^{-1}$) was observed.

### DISCUSSION

Of the various modalities used to treat PGL, surgical resection is the most commonly used initial treatment. The role of surgery in the management of PGL is to ensure an accurate histopathological diagnosis, reduce tumour bulk, relieve symptoms and prevent bleeding and perforation. Although the actual frequency of chemotherapy-related bleeding or perforation is not clear, the frequency of these complications in unresected patients with gastrointestinal lymphoma reported previously varies from 0% to 20% (Brooks et al, 1983; Gobbi et al, 1984; Rosenfelt et al, 1980). Another important aspect of surgery is tumour staging, and many authors have reported that this is the most important prognostic factor affecting survival (Lim et al, 1977; Shiu et al, 1982; Brooks et al, 1983; Dragosics et al, 1985; Hockey et al, 1987) Any additional treatment is based on the pathological stage, and surgical exploration with gastrectomy is the only means of achieving this.

Favourable results for stage I and II PGL using surgical resection followed by chemotherapy have been reported by several authors (Paulson et al, 1983; Sheridan et al, 1985; Shepherd et al, 1988; Bellesi et al, 1989; Pasini et al, 1994), but these reports failed to discriminate between stage I and stage II disease. In the present study, we focused on stage II PGL because the results obtained from a previous study (Takenaka et al, 1981) suggested that: (a) stage I PGL is cured by gastrectomy alone; and (b) stage II PGL relapses occasionally without adjuvant chemotherapy.

In our hospital (during the same period as our stage II study), 27 consecutive stage I PGL patients were treated by total gastrectomy alone. After 14–205 (median 99) months of follow-up, none has shown any sign of relapse (unpublished data). These results concur with the hypothesis that post-operative chemotherapy is not necessary for stage I PGL patients (Takenaka et al, 1981; Paulson et al, 1983).

Discrimination between stage $II_1$ and stage $II_2$ using Musshoff's staging system (Musshoff, 1977) was difficult in this study, because established staging procedures were not used. However, the results of the present study show that stage II PGL is curable with well-planned chemotherapy after surgical resection. Considering that a definite prognostic difference exists between stage $II_1$ and stage $II_2$ patients (Dragosics et al, 1984), further study is necessary to develop the best treatment modalities for stage $II_1$ and stage $II_2$ determined by established staging procedures.

The present adjuvant chemotherapy regimen consisting of post-operative chemotherapy with m-VEPA and consolidation chemotherapy with VQEP or VEMP requires about 2 years for completion. It is important to determine whether consolidation chemotherapy is necessary for the prevention of recurrence. No relapses were observed in the present study between 20 and 49 months among the five patients who refused consolidation chemotherapy. Adjuvant chemotherapy regimens for PGL stage II patients should be studied further and selected with consideration of both treatment duration and effectiveness. Considering a previous report on aggressive lymphoma (Fisher et al, 1993), we suggest that the standard CHOP regimen is a valid form of chemotherapy. We began an adjuvant chemotherapy study of PGL stage II patients using a CHOP regimen in November 1994. Severe toxicities and relapses have not been observed to date.

To preserve the stomach, patients with localized PGL have been treated with chemotherapy, radiotherapy and chemotherapy plus radiotherapy. Burgers et al (1988) reported a 4-year disease-free survival rate of 83% in 24 patients with stage I PGL treated with whole abdominal radiotherapy with a gastric bed boost. Maor et al (1990) reported a 5-year disease-free survival rate of 62% in 34 patients with stage I and II PGL treated with combination chemotherapy and involved-field radiotherapy. These results are not superior to our survival rate and disease-free survival rate obtained by surgery plus adjuvant chemotherapy. Although combination chemotherapy as an initial treatment has been shown to be useful in PGLs (Maor et al, 1984; Salles et al, 1991), data are not available concerning the treatment of localized PGL with chemotherapy alone.

*Helicobacter pylori* is present in 92% of gastric low-grade MALT lymphomas (Wotherspoon et al, 1991). After the eradication of *H. pylori* with antibiotics, five out of six low-grade gastric B-cell MALT lymphomas showed no evidence of lymphoma (Wotherspoon et al, 1993). However, the patients enrolled in the present study were not examined for *H. pylori* infection; that is a future task.

In addition, most low-grade MALT lymphomas are at stage IE; a minority are at stage $II_1$, according to Isaacson and Norton (1994). Only three (14%) of our stage II patients had low-grade MALT lymphoma. Interestingly, of our 22 stage I patients re-evaluated histologically, 16 cases were classified as low-grade MALT lymphoma, two as high-grade and four as non-MALT lymphoma (unpublished data).

According to our experience, about half of the localized PGL cases are stage I and the other half are stage II. If an accurate discrimination among stage I, stage $II_1$ and stage $II_2$ is possible by non-invasive or non-surgical procedures, e.g. endoscopic ultrasonography or CT, radiotherapy instead of surgery for stage I and chemotherapy for stage $II_1$ and stage $II_2$ may be indicated as an initial induction therapy.

Localized PGL should be studied prospectively regarding the relationships among histological grade, infection of *H. pylori*, treatment modality and prognosis.

Although our treatment results for stage II PGL should be assessed by multicentre prospective studies, we conclude for the present that patients with localized PGL should undergo surgical resection if possible, and, for patients found to have stage II disease, adjuvant chemotherapy offers the best chance of cure or long-term survival.

### ACKNOWLEDGEMENTS

This work was supported by Grants-in-Aid for Cancer Research from the Japanese Ministry of Health and Welfare and by the Foundation for the Promotion of Cancer Research.

### REFERENCES

Bellesi G, Alterini R, Meesori A, Bosi A, Bernardi F, Di Lollo S and Ferrini PR (1989) Combined surgery and chemotherapy for the treatment of primary gastrointestinal intermediate- or high-grade non-Hodgkin's lymphomas. *Br J Cancer* **60**: 244–248

Brooks JJ and Enterline HT (1983) Primary gastric lymphomas, a clinico-pathologic study of 58 cases with long-term follow-up and literature review. *Cancer* **51**: 701–711

© Cancer Research Campaign 1997

Burgers JMV, Taal BG, Van Heerge P, Somers R, Den Hartog Jager FCA and Hart AAM (1988) Treatment results of primary stage I and II non-Hodgkin's lymphoma of the stomach. *Radiother Oncol* **11:** 319–326

Carbone PP, Kaplan HS, Musshoff K, Smithers DW and Tubiana M (1971) Report of the committee on Hodgkin's disease staging classification. *Cancer Res* **31:** 1860–1861

Dawson IMP, Cornes JS and Morson BC (1961) Primary malignant lymphoid tumors of the intestinal tract, report of 37 cases with a study of factors influencing prognosis. *Br J Surg* **49:** 80–89

Dragosics B, Bauer P and Radaszkiewicz T (1985) Primary gastrointestinal non-Hodgkin's lymphomas. A retrospective clinicopathologic study of 150 cases. *Cancer* **55:** 1060–1073

Fisher RI, Gaynor ER, Dahlberg S, Oken MM, Grogan TM, Mize EM, Glick JH, Coltman CA and Miller TP (1993) Comparison of a standard regimen (CHOP) with three intensive chemotherapy regimens for advanced non-Hodgkin's lymphoma. *N Engl J Med* **328:** 1002–1006

Freeman G, Berg JW and Culter SJ (1972) Occurrence and prognosis of extranodal lymphomas. *Cancer* **29:** 252–260

Gobbi PG, Dionigi P, Barfieri F, Corbella F, Bertoloni D, Grignani G, Jemos V, Prieresca C and Ascari E (1990) The role of surgery in the multimodal treatment of primary gastric non-Hodgkin's lymphomas, a report of 76 cases and review of the literature. *Cancer* **65:** 2528–2536

Gospodarowicz MK, Bush RS, Brown TC and Chua T (1983): Curability of gastrointestinal lymphoma with combined surgery and radiation. *Int J Radiat Oncol Biol Phys* **9:** 3–9

Hockey MS, Powell J, Crocker J and Fielding JWL (1987) Primary gastric lymphoma. *Br J Surg* **74:** 483–487

Isaacson PG and Norton AJ (1994) *Extranodal Lymphomas*. Churchill Livingstone: Edinburgh

Isaacson PG and Spencer J (1987) Malignant lymphoma of mucosa-associated lymphoid tissue. *Histopathology* **11:** 445–462

Isaacson PG and Wright DH (1983) Malignant lymphoma of mucosa-associated tissue. A distinctive type of B-cell lymphoma. *Cancer* **52:** 1410–1416

Kaplan EL and Meier P (1958) Nonparametric estimation from incomplete observation. *J Ann Stat Assoc* **53:** 457–481

Lim FE, Hartman AS, Tan EGC, Cady B and Meissner W (1977) Factors in the prognosis of gastric lymphoma. *Cancer* **39:** 1715–1720

Lymphoma Study Group (1979) Combination chemotherapy with vincristine, cyclophosphamide (Endoxan), prednisolone and adriamycin (VEPA) in advanced adult non-Hodgkin's lymphoid malignancies; relation between T-cell or B-cell phenotype and response. *Jpn J Clin Oncol* **9** (suppl): 397–406

McKelvey EM, Gottlieb JA, Wilson HE, Haut A, Talley RW, Stephens R, Lane M, Gamble JF, Jones SE, Grozea PN, Gutterman J, Coltman C and Moon TE (1976) Hydroxydaunomycin (Adriamycin) combination chemotherapy in malignant lymphoma. *Cancer* **38:** 1484–1493

Maor MH, Maddux B, Osborne BM, Fuller LM, Sullivan JA, Nelson RS, Martin RG, Libshitz HI, Velasquez WS and Bennet RW (1984) Stage I E and stage II E non-Hodgkin's lymphomas of the stomach. Comparison of treatment modalities. *Cancer* **54:** 2330–2337

Maor MH, Velasquez WS, Fuller LM and Silvermintz KB (1990) Stomach conservation in stage I E and II E gastric non-Hodgkin's lymphoma. *J Clin Oncol* **8:** 266–271

Musshoff K (1977) Klinishe Stadieneinteilung der Nicht-Hodgkin-Lymphome. *Strahlentherapie* **153:** 218–221

Pasini F, Ambrosetti A, Sabbioni R, Todeschini G, Santo A, Meneghini V, Perona G and Cetto GL (1994) Postoperative chemotherapy increases the disease-free survival rate in primary gastric lymphomas stage I E and II E. *Eur J Cancer* **30A** (1): 33–36

Paulson S, Sheehan RG, Stone MJ and Frenkel EP (1983) Large cell lymphomas of the stomach: improved prognosis with complete resection of all intrinsic gastrointestinal disease. *J Clin Oncol* **1:** 263–269

Rodriguez V, Cabanillas F, Burgess MA, McKelvey EM, Valdivieso M, Bodey GP and Freireich EJ (1977) Combination chemotherapy ("CHOP-Bleo") in advanced (non-Hodgkin) malignant lymphoma. *Blood* **49:** 325–333

Rosenfelt R and Rosenberg SA (1980) Diffuse histiocytic lymphoma presenting with gastrointestinal tract lesions, the Stanford experience. *Cancer* **45:** 2188–2193

Sakai Y (1976) Treatment of malignant lymphoma (in Japanese). *Medicina* **13:** 1258–1263

Salles G, Herbrecht R, Tilly H, Berger F, Brousse N, Gisselbrecht C and Coiffier B (1991) Aggressive primary gastrointestinal lymphomas: review of 91 patients treated with the LNH-84 regimen. A study of the Groupe d'Etude des Lymphomes Agressifs. *Am J Med* **90:** 77–84

Shepherd FA, Evans WK, Kutas G, Yau JC, Dang P, Scott G, Farquharson A, Francombe WH, Bailey D and Baker MA (1988) Chemotherapy following surgery for stages I E and II E non-Hodgkin's lymphomas of the gastrointestinal tract. *J Clin Oncol* **6:** 253–260

Sheridan WP, Medley G and Brodie GN (1985) Non-Hodgkin's lymphoma of the stomach: a prospective pilot study of surgery plus chemotherapy in early and advanced disease. *J Clin Oncol* **3:** 495–500

Shimoyama M, Ota K, Kikuchi M, Yunoki K, Konda S, Takatsuki K, Ichimaru M, Ogawa M, Kimura I, Tominaga S, Tsugane S, Taguchi H, Minato K, Takenaka T, Tobinai K, Kurita S, Oyama A, Hisano S, Kozuru M, Matsumoto M, Nomura K, Takiguchi T, Sugai S, Yamaguchi K, Hattori T, Kinoshita K, Tajima K and Suemasu K for the Lymphoma Study Group (1981–1983) (1988) Chemotherapeutic results and prognostic factors of patients with advanced non-Hodgkin's lymphoma treated with VEPA or VEPA-M. *J Clin Oncol* **6:** 128–141

Shiu MH, Karas M, Nisce L, Lee BJ, Filippa DA and Lieberman PH (1982) Management of primary gastric lymphoma. *Ann Surg* **195:** 196–202

Skarin AT, Rosenthal DS, Moloney WC and Frei E III (1977) Combination chemotherapy of advanced non-Hodgkin lymphoma with bleomycin, adriamycin, cyclophosphamide, vincristine and prednisone (BACOP). *Blood* **49:** 759–770

Taal BG, Burgers JMV, Van Heerde P, Hart AAM and Somers R (1993) The clinical spectrum and treatment of primary non-Hodgkin's lymphoma of the stomach. *Ann Oncol* **4:** 839–846

Takenaka T, Konda C, Sakano T, Shimoyama M, Kitahara T, Minato K, Kitaoka H, Hirota T and Itabashi M (1981) On the significance of multiagent combination chemotherapy for primary gastric malignant lymphoma (in Japanese, summary with English). *J Jpn Soc Cancer Ther* **16:** 1310–1316

Takenaka T, Konda C, Sakano T, Shimoyama M, Kitahara T, Minato K and Kitaoka H (1982) Combination chemotherapy of primary gastric lymphoma with vincristine, Endoxan (cyclophosphamide), prednisolone and adriamycin (VEPA) (in Japanese, summary with English). *Cancer and Chemotherapy* **9:** 323–329

The Non-Hodgkin's Lymphoma Pathologic Classification Project (1982) National Cancer Institute sponsored study of classification of non-Hodgkin's lymphoma; summary and description of a working formulation for clinical usage. *Cancer* **49:** 2112–2135

Tondini C, Giardini R, Bozzetti F, Valagussa P, Santro A, Bertulli R, Balzarotti M, Rocca A, Lombardi F, Ferreri AJM and Bonadonna G (1993) Combined modality treatment for primary gastrointestinal non-Hodgkin's lymphoma: The Milan Cancer Institute Experience. *Ann Oncol* **4:** 831–837

Wotherspoon AC, Ortiz-Hidalgo, Falzon MF and Isaacson PG (1991) *Helicobacter pylori*-associated gastritis and primary B-cell gastric lymphoma. *Lancet* **338:** 1175–1176

Wotherspoon AC, Doglioni C, Diss TC, Pan L, Moschini A, Boni M and Isaacson PG (1993) Regression of primary low-grade B-cell gastric lymphoma of mucosa-associated lymphoid tissue type eradication of *Helicobacter pylori*. *Lancet* **342:** 575–577

© Cancer Research Campaign 1997


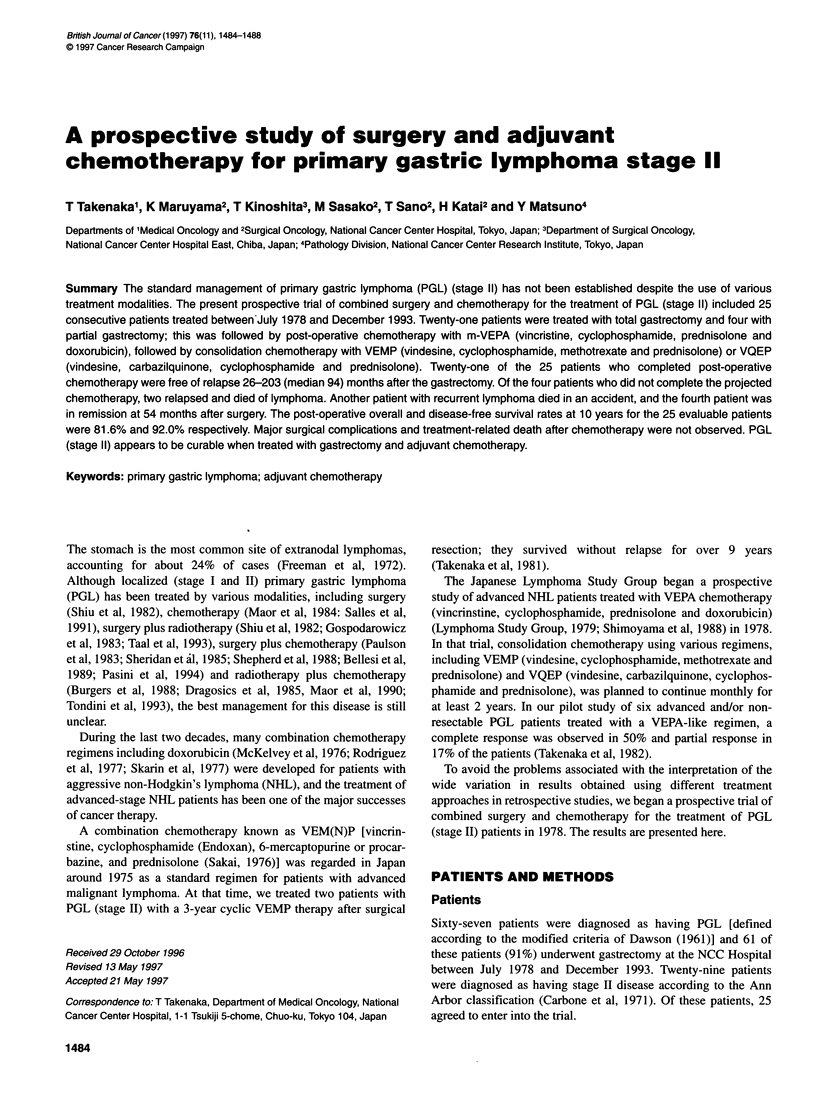

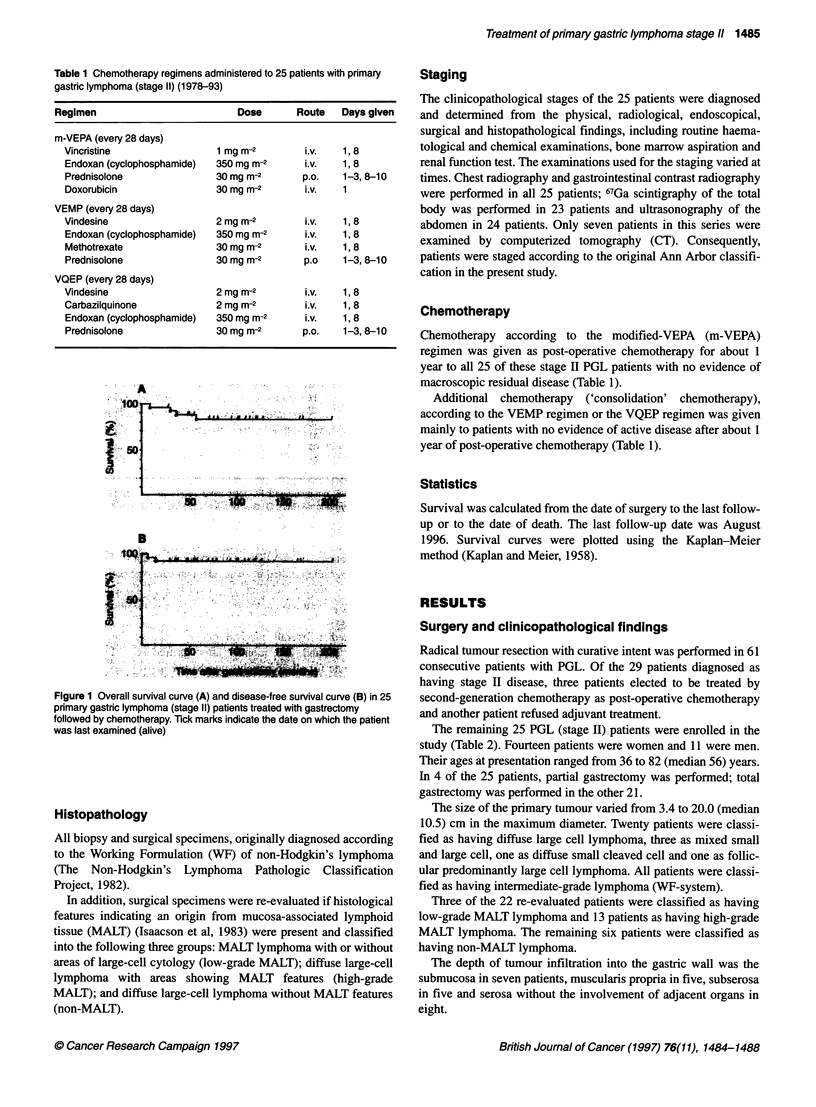

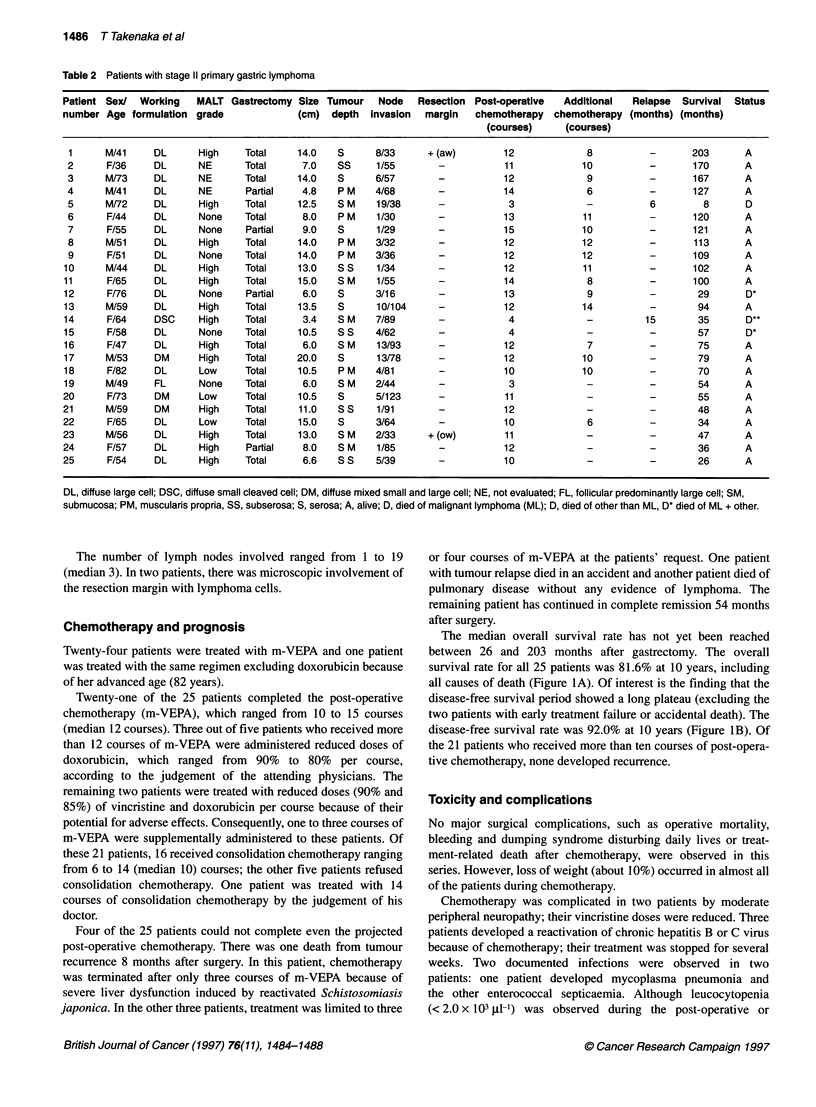

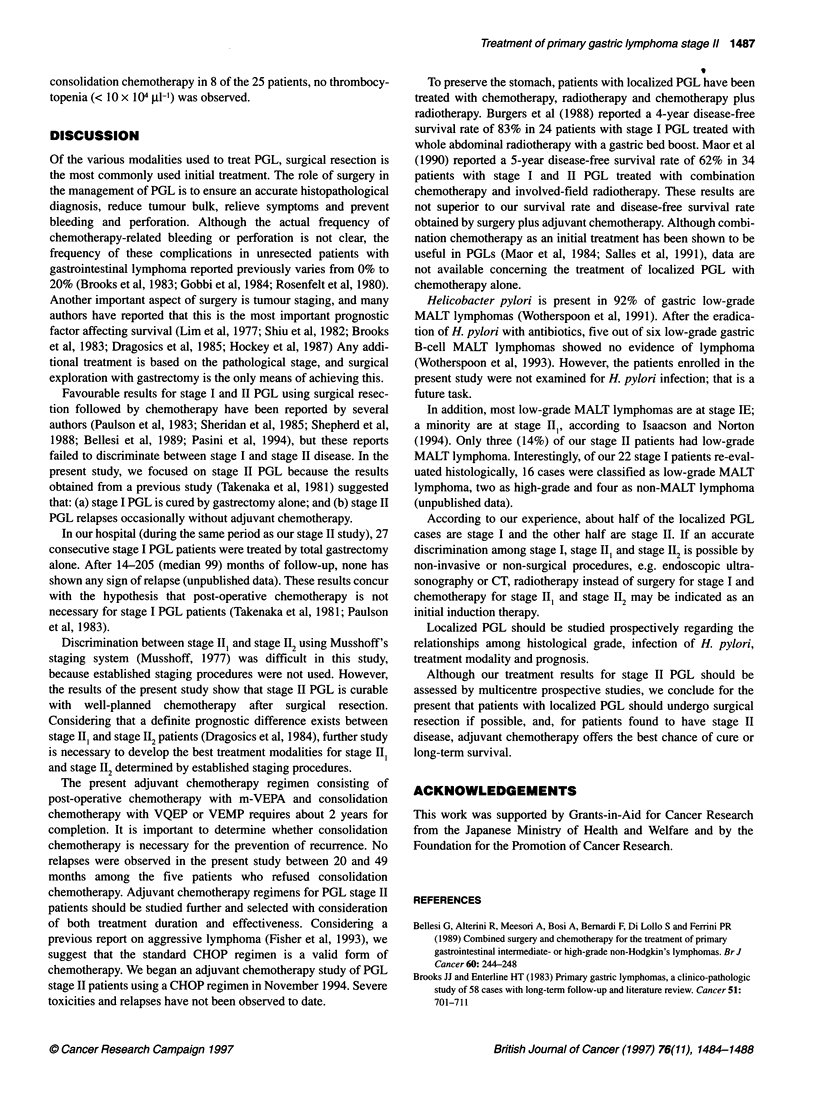

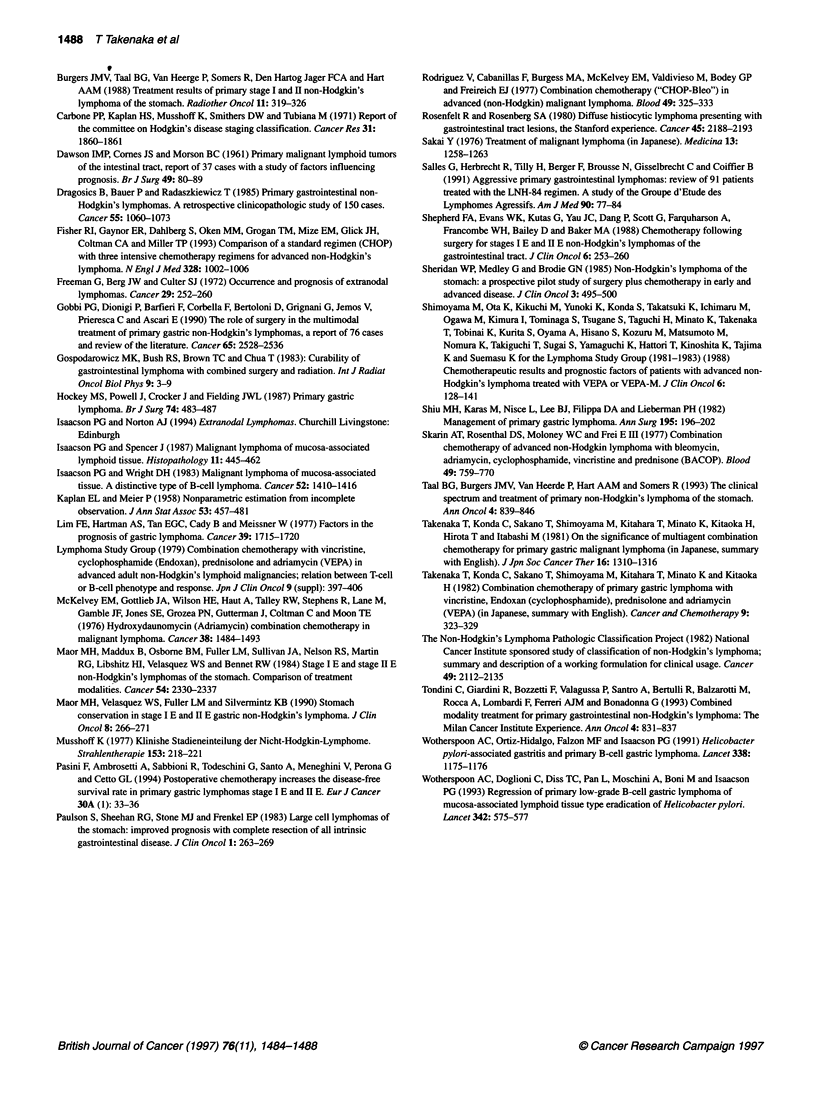


## References

[OCR_00529] Bellesi G., Alterini R., Messori A., Bosi A., Bernardi F., di Lollo S., Ferrini P. R. (1989). Combined surgery and chemotherapy for the treatment of primary gastrointestinal intermediate- or high-grade non-Hodgkin's lymphomas.. Br J Cancer.

[OCR_00536] Brooks J. J., Enterline H. T. (1983). Primary gastric lymphomas. A clinicopathologic study of 58 cases with long-term follow-up and literature review.. Cancer.

[OCR_00547] Burgers J. M., Taal B. G., van Heerde P., Somers R., den Hartog Jager F. C., Hart A. A. (1988). Treatment results of primary stage I and II non-Hodgkin's lymphoma of the stomach.. Radiother Oncol.

[OCR_00552] Carbone P. P., Kaplan H. S., Musshoff K., Smithers D. W., Tubiana M. (1971). Report of the Committee on Hodgkin's Disease Staging Classification.. Cancer Res.

[OCR_00557] DAWSON I. M., CORNES J. S., MORSON B. C. (1961). Primary malignant lymphoid tumours of the intestinal tract. Report of 37 cases with a study of factors influencing prognosis.. Br J Surg.

[OCR_00562] Dragosics B., Bauer P., Radaszkiewicz T. (1985). Primary gastrointestinal non-Hodgkin's lymphomas. A retrospective clinicopathologic study of 150 cases.. Cancer.

[OCR_00567] Fisher R. I., Gaynor E. R., Dahlberg S., Oken M. M., Grogan T. M., Mize E. M., Glick J. H., Coltman C. A., Miller T. P. (1993). Comparison of a standard regimen (CHOP) with three intensive chemotherapy regimens for advanced non-Hodgkin's lymphoma.. N Engl J Med.

[OCR_00573] Freeman C., Berg J. W., Cutler S. J. (1972). Occurrence and prognosis of extranodal lymphomas.. Cancer.

[OCR_00577] Gobbi P. G., Dionigi P., Barbieri F., Corbella F., Bertoloni D., Grignani G., Jemos V., Pieresca C., Ascari E. (1990). The role of surgery in the multimodal treatment of primary gastric non-Hodgkin's lymphomas. A report of 76 cases and review of the literature.. Cancer.

[OCR_00589] Hockey M. S., Powell J., Crocker J., Fielding J. W. (1987). Primary gastric lymphoma.. Br J Surg.

[OCR_00593] Isaacson P. G., Spencer J. (1987). Malignant lymphoma of mucosa-associated lymphoid tissue.. Histopathology.

[OCR_00601] Isaacson P., Wright D. H. (1983). Malignant lymphoma of mucosa-associated lymphoid tissue. A distinctive type of B-cell lymphoma.. Cancer.

[OCR_00608] Lim F. E., Hartman A. S., Tan E. G., Cady B., Meissner W. A. (1977). Factors in the prognosis of gastric lymphoma.. Cancer.

[OCR_00625] Maor M. H., Maddux B., Osborne B. M., Fuller L. M., Sullivan J. A., Nelson R. S., Martin R. G., Libshitz H. I., Velasquez W. S., Bennett R. W. (1984). Stages IE and IIE non-Hodgkin's lymphomas of the stomach. Comparison of treatment modalities.. Cancer.

[OCR_00631] Maor M. H., Velasquez W. S., Fuller L. M., Silvermintz K. B. (1990). Stomach conservation in stages IE and IIE gastric non-Hodgkin's lymphoma.. J Clin Oncol.

[OCR_00619] McKelvey E. M., Gottlieb J. A., Wilson H. E., Haut A., Talley R. W., Stephens R., Lane M., Gamble J. F., Jones S. E., Grozea P. N. (1976). Hydroxyldaunomycin (Adriamycin) combination chemotherapy in malignant lymphoma.. Cancer.

[OCR_00636] Musshoff K. (1977). Klinische Stadieneinteilung der Nicht-Hodgkin-Lymphome. Strahlentherapie.

[OCR_00640] Pasini F., Ambrosetti A., Sabbioni R., Todeschini G., Santo A., Meneghini V., Perona G., Cetto G. L. (1994). Postoperative chemotherapy increases the disease-free survival rate in primary gastric lymphomas stage IE and IIE.. Eur J Cancer.

[OCR_00646] Paulson S., Sheehan R. G., Stone M. J., Frenkel E. P. (1983). Large cell lymphomas of the stomach: improved prognosis with complete resection of all intrinsic gastrointestinal disease.. J Clin Oncol.

[OCR_00651] Rodriguez V., Cabanillas F., Burgess M. A., McKelvey E. M., Valdivieso M., Bodey G. P., Freireich E. J. (1977). Combination chemotherapy ("CHOP-Bleo") in advanced (non-Hodgkin) malignant lymphoma.. Blood.

[OCR_00656] Rosenfelt F., Rosenberg S. A. (1980). Diffuse histiocytic lymphoma presenting with gastrointestinal tract lesions. The Stanford experience.. Cancer.

[OCR_00663] Salles G., Herbrecht R., Tilly H., Berger F., Brousse N., Gisselbrecht C., Coiffier B. (1991). Aggressive primary gastrointestinal lymphomas: review of 91 patients treated with the LNH-84 regimen. A study of the Groupe d'Etude des Lymphomes Agressifs.. Am J Med.

[OCR_00669] Shepherd F. A., Evans W. K., Kutas G., Yau J. C., Dang P., Scott J. G., Farquharson H. A., Francombe W. H., Bailey D., Baker M. A. (1988). Chemotherapy following surgery for stages IE and IIE non-Hodgkin's lymphoma of the gastrointestinal tract.. J Clin Oncol.

[OCR_00675] Sheridan W. P., Medley G., Brodie G. N. (1985). Non-Hodgkin's lymphoma of the stomach: a prospective pilot study of surgery plus chemotherapy in early and advanced disease.. J Clin Oncol.

[OCR_00692] Shiu M. H., Karas M., Nisce L., Lee B. J., Filippa D. A., Lieberman P. H. (1982). Management of primary gastric lymphoma.. Ann Surg.

[OCR_00696] Skarin A. T., Rosenthal D. S., Moloney W. C., Frei E. (1977). Combination chemotherapy of advanced non-Hodgkin lymphoma with bleomycin, adriamycin, cyclophosphamide, vincristine, and prednisone (BACOP).. Blood.

[OCR_00703] Taal B. G., Burgers J. M., van Heerde P., Hart A. A., Somers R. (1993). The clinical spectrum and treatment of primary non-Hodgkin's lymphoma of the stomach.. Ann Oncol.

[OCR_00708] Takenaka T., Konda C., Sakano T., Shimoyama M., Kitahara T., Minato K., Kitaoka H., Hirota T., Itabashi M. (1981). [On the significance of multiagent combination chemotherapy for primary gastric malignant lymphoma (author's transl)].. Nihon Gan Chiryo Gakkai Shi.

[OCR_00714] Takenaka T., Konda C., Sakano T., Shimoyama M., Kitahara T., Minato K., Kitaoka H. (1982). [Combination chemotherapy of primary gastric lymphoma with vincristine, endoxan (cyclophosphamide), prednisolone and adriamycine (VEPA)].. Gan To Kagaku Ryoho.

[OCR_00728] Tondini C., Giardini R., Bozzetti F., Valagussa P., Santoro A., Bertulli R., Balzarotti M., Rocca A., Lombardi F., Ferreri A. J. (1993). Combined modality treatment for primary gastrointestinal non-Hodgkin's lymphoma: the Milan Cancer Institute experience.. Ann Oncol.

[OCR_00740] Wotherspoon A. C., Doglioni C., Diss T. C., Pan L., Moschini A., de Boni M., Isaacson P. G. (1993). Regression of primary low-grade B-cell gastric lymphoma of mucosa-associated lymphoid tissue type after eradication of Helicobacter pylori.. Lancet.

[OCR_00735] Wotherspoon A. C., Ortiz-Hidalgo C., Falzon M. R., Isaacson P. G. (1991). Helicobacter pylori-associated gastritis and primary B-cell gastric lymphoma.. Lancet.

